# Clinical and functional characteristics of OSA in children with comorbid asthma treated by leukotriene receptor antagonist: A descriptive study

**DOI:** 10.3389/fneur.2022.1065038

**Published:** 2023-01-04

**Authors:** Sy Duong-Quy, Yen Nguyen-Hoang, Le Nguyen-Ngoc-Quynh, Mai Nguyen-Thi-Phuong, Hanh Nguyen-Thi-Bich, Huong Le-Thi-Minh, Thuy Nguyen-Thi-Dieu

**Affiliations:** ^1^Biomedical Research Centre, Lam Dong Medical College, Dalat, Vietnam; ^2^Division of Immuno-Allergology, Penn State Medical College, Hershey Medical Center, Hershey, PA, United States; ^3^Department of Outpatient, Pham Ngoc Thach University of Medicine, Ho Chi Minh City, Vietnam; ^4^Department of Pediatrics, Lac Viet Friendly Hospital, Vinh Yên, Vinh Phuc, Vietnam; ^5^Department of Immuno-Allergology, Asthma and Rheumatology, National Children's Hospital, Hanoi, Vietnam; ^6^Pediatric Centre, Vinmec Times City International Hospital, Hanoi, Vietnam; ^7^Department of Pediatrics, Hanoi Medical University, Hanoi, Vietnam

**Keywords:** asthmatic children, OSA, apnea-hypopnea index, snoring, leukotriene receptor antagonists

## Abstract

**Background:**

Obstructive sleep apnea (OSA) is the most common form of respiratory disorders during sleep in children, especially those with severe asthma. However, optimal treatment of asthma might significantly improve OSA severity.

**Methods:**

It was a cohort study including children aged >5 years old and diagnosed with asthma according to GINA (Global Initiative for Asthma). The data related to age, gender, height, weight, body mass index (BMI), clinical symptoms and medical history of asthma, spirometry (FEV_1_: forced expiratory in 1 s), and exhaled nitric oxide (F_E_NO) were recorded for analysis. Respiratory polygraphy (RPG) was done for each study subject to diagnose OSA and its severity.

**Results:**

Among 139 asthmatic children, 99 patients with OSA (71.2%) were included in the present study (9.3 ± 0.2 years): 58.6% with uncontrolled asthma and 32.3% with partial controlled asthma. The mean ACT (asthma control testing) score was 19.0 ± 3.4. The most frequent night-time symptoms were restless sleep (76.8%), snoring (61.6%), sweating (52.5%), and trouble breathing during sleep (48.5%). The common daytime symptoms were irritable status (46.5%) and abnormal behavior (30.3%). The mean AHI (apnea-hypopnea index) was 3.5 ± 4.0 events/h. There was a significant correlation between BMI and snoring index (*R* = 0.189 and *P* = 0.027), bronchial and nasal F_E_NO with AHI (*R* = 0.046 and *P* < 0.001; *R* = 0.037 and *P* < 0.001; respectively). There was no significant correlation between asthma level, FEV_1_ and AHI. The severity of asthma and respiratory function were improved significantly after 3 months and 6 months of asthma treatment in combination with leukotriene receptor antagonist (LRA) treatment. The symptoms related to OSA were significantly improved after treatment with LRA. The severity of OSA was decreased significantly after 3 months and 6 months of treatment.

**Conclusion:**

The treatment of asthmatic children with comorbid OSA by LRA in combination with standard therapy for asthma could improve the control of asthma and the symptoms and severity of OSA.

## Introduction

Obstructive sleep apnea (OSA) is a continuous repetition of partial or complete obstruction of the upper airway during sleep resulting in hypopnea or total apnea despiting respiratory efforts (1). The incidence of OSA in children has been estimated about 2% (1–5%) (2). OSA has been found at any age with the highest age of 2–8 years and higher in male than female, and in Asians children (2, 3).

OSA and asthma are two co-diseases, both of which share the same symptoms because they are associated with airflow limits and increased respiratory exertion, as a result of narrowing the airways during sleeping (4). In patients with asthma, OSA plays a role as a harmful contributing factor for worsening asthma due to sleep disturbance, decreased sleep quality, destabilized bronchial tonus during sleep, increased microarousal and wake-up, and daytime sleepiness (5). Increased abdominal pressure during OSA period also contributes to gastroesophageal reflux, increased reactivity of the bronchi inducing bronchial hyperreactivity (6). Patients with hard-to-control asthma may have an increase in the number of stages with OSA and a decrease in blood oxygen saturation, especially during sleep phases with rapid eye movement (7). In children, OSA causes nocturnal intermittent hypoxia due to apnea and hypopnea which is the cause of pathogenetic disorders. These mechanism consequences impact on hemodynamics and metabolism, increasing the risk of cardiovascular diseases such as heart failure, high blood pressure, and pulmonary hypertension (8). Moreover, OSA may give serious consequences on the mental, motor, and physical development of children because it affects directly on the process of physical and psychological development. It induces cognitive impairment, lack of concentration, decreased learning and memory ability (9).

Currently, the main treatments of OSA in children is adenotonsillectomy. However, this intervention is an invasive method for children with asthma (1). Some recent reported showed that tonsillar tissue from children with OSA may overexpress cysteine leukotriene receptor-1 (CysLT1), which can be treated with anti-inflammatory therapy such as leukotriene receptor antagonists (10, 11). In children with non-severe OSA, this therapy might have the potential efficacy in the treatment of asthmatic children with OSA associated with adenotonsillar hypertrophy (11, 12).

Therefore, the present study was realized to describe the clinical and functional characteristics of OSA in children with asthma and the clinical efficacy of antileukotriene drugs in the treatment of asthma and OSA in these children.

## Methods

### Subjects

There were 99 patients over 5 years old who were diagnosed and treated with asthma in the Department of Immuno-Allergology, Asthma and Rheumatology of the National Children's Hospital from January 2016 to January 2019. The present study was approved by the Ethics Council in Biomedical Research of Hanoi Medical University within the decision No. 187/HDDD/DHY-HN.

#### Inclusion criteria

Children having the following criteria were included in the present study: aged >5 years-old, diagnosed with asthma according to GINA and without acute asthma attacks; being able to do the required tests; the agreement and written consent were obtained from patients or their guardians.

#### Exclusion criteria

Children having one of the following criteria were excluded from the study: having an acute respiratory infection; being unable to perform the laboratory testings; patients with other acute or chronic diseases (heart failure, renal failure, or mental disorders); patients with current treatment of corticosteroids (oral or intranasal); patients with oral antihistamine preparations or nasal decongestants; patients with adenotonsillar hypertrophy.

### Study design

**Figure 1:**
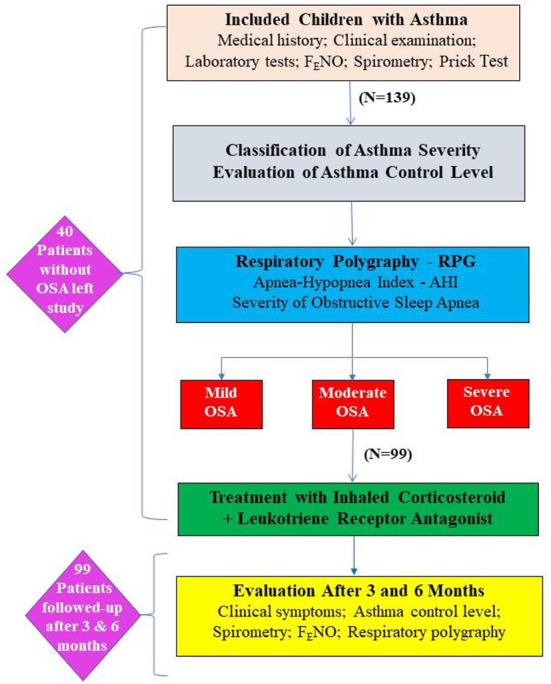
Flowchart of the study method. AHI, apnea-hypopnea index; OSA, obstructive sleep apnea; RPG, respiratory polygraphy.

It was a cross-sectional and cohort study, including asthmatic children >5 years who were followed-up in the Department of Immuno-Allergology, Asthma and Rheumatology of the National Children's Hospital, Hanoi – Vietnam. Asthmatic childrens received clinical examination, biology tests, skin prick-test, lung function testing with spirometry and respiratory polygraphy (Figure 1). They were then classified as mild, moderate or severe OSA based on the apnea – hypopnea index (AHI) and treated with inhaled corticosteroid (ICS) plus leukotrien receptor antagonist (LRA).

### Asthma evaluation

The diagnosis of asthma was based on the criteria recommended by Global Initiative for Asthma (GINA) 2015 for children over 5 years old (13). Depending on asthma severity, the study subjects were treated as recommended by GINA (13). The asthma control test (ACT) was used as a self-assessment by the study subjects (≥12 years) or their parents (<12 years). The level of asthma control was defined as recommended by GINA: controlled, partially controlled, and uncontrolled asthma (13).

### Lung function testing (LFT)

LFT (*spirometry*) was done by Koko (nSpire Health, Inc., Longmont, CO, USA). The reversibility of forced expiratory volume in one second (FEV_1_) was evaluated after using 200 μg of salbutamol for 15 min. The test was positive when there was the increase of FEV_1_ ≥12% and >200 mL (14). Measuring exhaled NO concentration was done by Hypair FeNO+ Device (Medisoft; Sorinnes, Belgium) with expiratory air flow of 50, 100, 150, and 350 mL/s. The fractional exhaled nitric oxide (F_E_NO) levels were classified as recommended by the American Thoracic Society/European Respiratory Society (ATS/ERS) for children (<20 ppb: normal, 20–35 ppb: increased, and >35 ppb: highly increased) (15). The level of alveolar concentration of exhaled NO (C_A_NO) <5 ppb was defined as normal (15).

### Skin prick test (SPT)

The SPT was done by using Stallergenes Kit (Stallergenes; London, UK), and the negative control was 0.9% saline solution and the positive control was 1 mg/mL of histamine. Six respiratory allergens, including *Dermatophagoides pteronyssinus* (Dp), *Dermatophagoides farinae* (Df), *Blomia tropicalis* (Blo), and hairs and epidermis of dogs, cats, and cockroaches were tested. The SPT was considered positive when the wheal size exceeded the negative control by 3 mm (14).

### Respiratory polygraphy (RPG)

RPG was done with Apnea Link device (ResMed; San Diego, California, USA). OSA was defined with RPG by using AHI to classify the severity of OSA in children as recommended: normal (non-OSA): AHI <1/h; mild OSA: 1/h ≤ AHI ≤ 5/h; moderate OSA: 5/h <AHI ≤ 10/h; severe OSA: AHI >10/h (16).

### Data collection

All data related to age, gender, height, weight, BMI, medical and family history of asthma, clinical characteristics, LFT parameters (FEV_1_, FVC, FEV_1_/FVC, and PEF), exhaled NO (bronchial F_E_NO, nasal F_E_NO, and CANO), SPT and RPG parameters of the study subjects were collected for statistical analyses.

### Statistical analysis

SPSS 22.0 software (IBM Corporation, Armonk, NY, USA) was used to analyse the collected data. Continuous variables were presented as mean ± standard deviation (SD). Skewness-Kurtosis test was used for evaluating the normal distribution and Kruskal- Wallis test was done for performing the pairwise comparison. The regression analysis was used to measure the correlation between AHI and continuous variables, with the correlation coefficient *R* of Pearson for normal distribution variables and of Spearman for non-normal distribution variables. The Mann–Whitney *U*-test was used to evaluate the correlation between AHI and asthma severity.

## Results

### Clinical, biological and functional characteristics of study subjects

The results of the present study showed that among 139 asthmatic children there was only 99 patients with OSA were included for analysis. Among these 99 patients, male patients were accounted for 74.7% and its percentage was 2.8 times higher than female (Table 1). The mean age of study patients was 9.26 years (range: 5–15 years old) with the mean BMI of 17.4 kg/m^2^. Among these 99 asthmatic patients with OSA, there was 91.9% having the history of allergic conditions; of which, allergic rhinitis was the most common comorbidity (85.86%); 64.7% of them had siblings, parents or grandparents with allergic diseases (Table 1). There was 14.1% of study subjects had been diagnosed with gastroesophageal reflux. Approximately 44.4% of the study subjects had mild asthma, 10.1% with intermittent asthma, 41.41% with moderate asthma and 4.04% had severe asthma (Table 1). Among them, 58.59% of subjects were in uncontrolled asthma, 32.32% were in partially controlled asthma and 9.09% with controlled asthma. There were 61.6% of patients with mild OSA, 25.3% with moderate OSA, and 13.1% with severe OSA (AHI ≥10/h). The average AHI index was 3.45 ± 4.01/h (1–21/h) (Table 1).

**Table 1:** General characteristics of study subjects.

**Parameters (*N* = 99)**	**Values**
Age, years (mean ± SD)	9.26 ± 0.19
Female (Male), %	25.3 (74.7)
Height, cm	132.8 ± 1.13
Weight, kg	31.1 ± 0.85
BMI, kg/m^2^	17.4 ± 2.8
**Allergy status, %**	91.9
Eczema,%	34.3
Allergic rhinitis,%	85.8
Conjunctivitis,%	42.4
Drug allergies, %	3.0
Food allergies, %	13.1
Family history of allergy, %	64.7
Gastroesophageal reflux, %	14.1
**Asthma severity level**	
Intermittent, %	10.1
Mild, %	44.4
Moderate, %	41.4
Severe, %	4.0
**Level of asthma control**	
Total controlled, %	9.1
Partially controlled, %	32.3
Uncontrolled, %	58.5
ACT Score, mean ± SD	19.2 ± 3.4
**Peripheral blood count**	
White blood cells, number x10^3^/mm^3^	9.6 ± 2.9
Neutrophils, %	52.4 ± 1.5
Lymphocytes, %	33.1 ± 1.3
Eosinophils, %	6.4 ± 5.0
CRP mg/L, median (min-max)	3.8 (0 - 58.5)
IgE, UI/L	1,502 ± 1,300
**Skin prick test**	
Dp, %	67.7
Df, %	69.7
Blo, %	44.4
Cockroach, %	22.2
Dog hair, %	13.1
Cat hair, %	17.2
**Lung function testing**	
FEV1, % (mean ± SD)	85.1 ± 16.1
FVC, % (mean ± SD)	92.1 ± 15.0
FEV_1_/FVC, % (mean ± SD)	92.3 ± 12.6
PEF, % (mean ± SD)	68.9 ± 17.4
**Exhaled nitric oxide**	
Bronchial F_E_NO, ppb (mean ± SD)	22.1 ± 20.4
Nasal F_E_NO, ppb (mean ± SD)	1,505.9 ± 951.8
C_A_NO, ppb (mean ± SD)	7.4 ± 6.9
AHI, mean ± SD (min-max)	3.45 ± 4.01 (1–21)
Mild, %	61.6
Moderate, %	25.3
Severe, %	13.1

ACT, asthma control test; BMI, body mass index; AHI, apnea-hypopnea index; Blo, Blomia tropicalis; C_A_NO, alveolar concentration of nitric oxide; CRP, C reactive protein; Df, Dermatophagoides farinae; Dp, Dermatophagoides pteronyssinus; F_E_NO, fractional exhaled nitric oxide; FEV_1_, forced expiratory flow in 1 s; FVC, forced vital capacity; IgE, immunoglobulin E; PEF, peak expiratory flow; ppb, part per billion.

The majority of asthmatic children were allergic to house dust mites with 67.7% for *Dp*, 69.7% for *Df*, and 44.4% for *Blomia tropicalis* (Table 1). The mean levels of IgE and the percentage of eosinophils in the study subjects were higher than normal values (Table 1). The mean FEV_1_ and peak expiratory flow (PEF) in the study subjects were mildly lower than normal values, while other parameters (FEV_1_/FVC and FVC) were in normal range (Table 1). The nasal and bronchial F_E_NO were higher than normal values (1,505 ± 951.8 ppb and 22.1 ± 20.4 ppb; respectively; Table 1).

### Nocturnal and daytime symptoms and odds ratio of OSA in study subjects

The results showed the high percentages of asthmatic children with OSA who had snoring (61.6%), disturbed sleep (76.8%), and complaints with nocturnal sweats (52.5%). Parents also reported other symptoms: difficulty falling asleep (46.5%), difficulty breathing while sleeping (48.5%), frequently awake (38%), enuresis (11.1%) (Table 2). Asthmatic children who had snoring, disturbed sleep, and difficulty falling asleep were significantly higher odds ratio (OR) of OSA than those without these symptoms [OR = 3.75 (1.7–8.23) and *P* = 0.01; OR = 2.50 (1.1–5.67) and *P* = 0.028; OR = 2.44 (1.12–5.34) and *P* = 0.025; respectively; Table 2]. For daytime symptoms, asthmatic children with abnormal behavior had higher risk of OSA [OR = 3.04 (1.09 – 8.53) and *P* = 0.034; Table 2].

**Table 2:** Frequency and odds ratio of nocturnal and daytime symptoms of OSA in asthmatic children.

**Parameters (*N* = 99)**	**%**	**Odds ratio value (95% confidence interval)**	***P***
**Symptoms at night**	
Snoring	61.6	3.75 (1.70–8.23)	0.010
Difficulty for falling asleep	46.5	2.50 (1.10–5.67)	0.028
Difficulty breathing during sleep	48.5	1.41 (0.67–2.98)	0.365
Disturbed sleep	76.8	2.44 (1.12–5.34)	0.025
Frequent awake	38.3	1.45 (0.67–3.20)	0.352
Nocturnal sweating	52.5	1.22 (0.59–2.55)	0.592
Enuresis	11.1	0.88 (0.29–2.70)	0.816
**Daytime symptoms**	
Abnormal behavior	30.3	3.04 (1.09–8.53)	0.034
Irritable	46.5	1.80 (0.83–3.90)	0.134
Agitated	29.3	1.46 (0.6–3.37)	0.417
Sleepiness	26.3	2.5 (0.89–7.04)	0.085

### Correlation between asthma parameters, F_E_NO, and OSA severity measured by AHI

**Figure 2:**
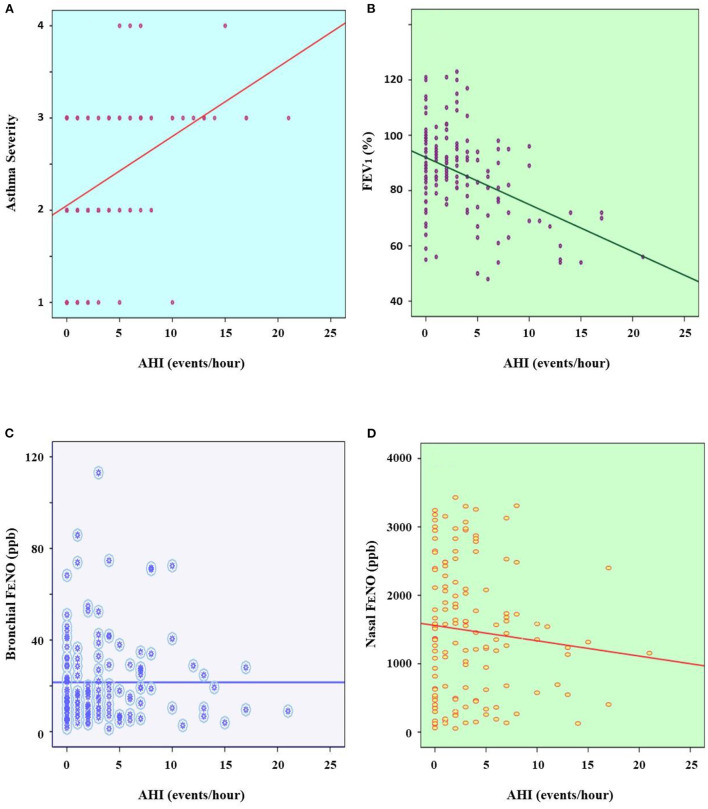
Correlation between asthma severity **(A)**, FEV_1_ **(B)**, bronchial F_E_NO **(C)**, and nasal F_E_NO **(D)** with AHI. AHI, apnea-hypopnea index; FEV_1_, forced expiratory volume in 1 s; F_E_NO, fractional exhaled nitric oxide; ppb, part per billion.

The results showed that there was no significant correlation between the level of asthma severity and AHI (*P* > 0.05; Figure 2A). There was no significant correlation between FEV_1_ and AHI (*P* > 0.05; Figure 2B). There was a significant and weak correlation between BMI and snoring in asthmatic children with OSA (*R* = 0.189 and *P* = 0.027). There were also the very weak correlations between bronchial F_E_NO and nasal F_E_NO with AHI (*R* = 0.046 and *P* = 0.001; *R* = 0.037 and *P* = 0.002; respectively; Figures 2C,D).

### Modification of clinical and functional parameters related to asthma after treatment

**Figure 3:**
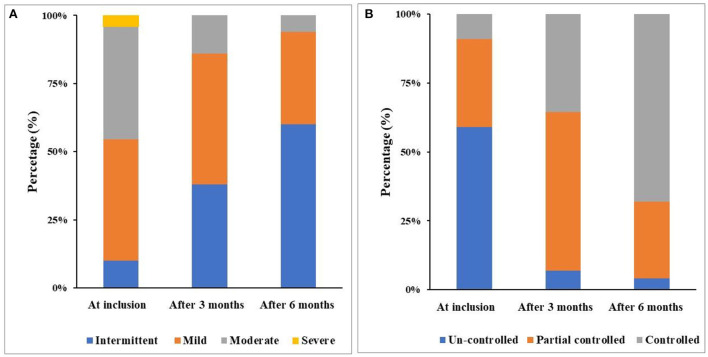
Distribution of asthma severity **(A)** and asthma control **(B)** after treatment.

The results showed that after treating with leukotriene receptor antagonists (LRA), the proportion of intermittent asthma increased from 10.1% at inclusion to 38.3% after 3 months and 60% after 6 months, while moderate and severe asthma decreased from 41.5 to 4.0% at inclusion to 6.1 and 0.0% after 6 months, respectively (Figure 3A). On the other hand, the percentage of un-controlled asthma and partial controlled asthma also decreased significantly (58.5 to 4.0% and 32.3 to 28.2%, respectively; Figure 3B). The percentage of totally controlled asthma increased from 9.1 to 35.5% after 3 months and 67.9% after 6 months (Figure 3B). ACT score increased from 19.2 ± 3.4 to 22.6 ± 4.7 points after 3 months and 24.2 ± 5.2 points after 6 months of treatment. The levels of FEV_1_, FVC, peak expiratory flow (PEF) and bronchial F_E_NO in the study subjects were improved markedly after 3 months and 6 months of treatment (Table 3).

**Table 3:** Modification of respiratory function and bronchial FENO after treatment.

**Patient characteristics (*N* = 99)**	**At inclusion**	**After 3 months**	**After 6 months**	***P***
FEV1, %	85.1 ± 16.1	94.3 ± 14.5	99.2 ± 16.7	0.032^*^; 0.004^**^; 0.067^***^
FVC, %	92.1 ± 15	99.3 ± 14.6	103.2 ± 12.7	0.058^*^; 0.037^**^; 0.076^***^
FEV1/FVC, %	92.3 ± 12.6	96.3 ± 13.5	96.0 ± 12.8	0.055^*^; 0.059^**^; 0.972^***^
PEF, %	68.9 ± 17.4	78.2 ± 16.3	80.1 ± 17.3	0.021^*^; 0.016^**^; 0.122^***^
Bronchial FENO, ppb	22.1 ± 20.4	15.1 ± 12.5	14.3 ± 11.6	0.019^*^; 0.014^**^; 0.233^***^
ACT score	19.2 ± 3.4	22.6 ± 4.7	24.2 ± 5.2	0.046^*^; 0.038^**^; 0.225^***^

ACT, asthma control test; FENO, fractional exhaled nitric oxide; FEV_1_, forced expiratory flow in 1 s; FVC, forced vital capacity; PEF, peak expiratory flow; ppb, part per billion. ^*^After 3 months vs. at inclusion; ^**^After 6 months vs. at inclusion; ^***^After 6 months vs. after 3 months.

### Modification of clinical and functional parameters related to OSA after treatment

The results of the present study showed that the symptoms related to OSA at night including snoring, difficulty falling asleep, difficulty breathing during sleep, disturbed sleep, frequent awake, nocturnal sweating, and enuresis were significantly improved after treatment (Table 4). The daytime symptoms due to the consequences of OSA were also improved markedly after treatment (Table 4). Agitation symptom was decreased from 29.3% at inclusion to 0.0% after 6 months of treatment; daytime sleepiness was dropped from 26.3% at inclusion to 3.8% after 6 months of treatment; abnormal behavior was decreased from 30.3% at inclusion to 9.4% after 6 months of treatment (Table 4).

**Table 4:** Modification of clinical symptoms related OSA after treatment.

**Patient characteristics (*N* = 99)**	**At inclusion % (*N*)**	**After 3 months % (*N*)**	**After 6 months % (*N*)**	***P***
**Symptoms at night**
Snoring	61.6 (62)	44.4 (44)	6.0 (6)	0.005^*^; <0.00001^**^; <0.00001^***^
Difficulty for falling asleep	46.5 (47)	18.1 (18)	11.1 (11)	<0.001^*^; <0.001^**^; 0.079^***^
Difficulty breathing during sleep	48.5 (48)	5.1 (5)	0.0 (0)	<0.00001^*^; <0.00001^**^; <0.00001^***^
Disturbed sleep	76.8 (76)	73.7 (73)	56.6 (55)	0.310^*^; <0.001^**^; <0.003^***^
Frequent awake	38.3 (38)	9.1 (9)	0.0 (0)	<0.00001^*^; <0.00001^**^; 0.001^***^
Nocturnal sweating	52.5 (52)	18.1 (18)	2.0 (2)	<0.00001^*^; <0.00001^**^; <0.0001^***^
Enuresis	11.1 (11)	0.0 (0)	0.0 (0)	<0.0001^*^; <0.0001^**^; N/A^***^
**Daytime symptoms**
Abnormal behavior	30.3 (30)	17.2 (17)	9.4 (9)	0.014^*^; <0.0001^**^; 0.046^***^
Irritable	47.4 (47)	37.3 (37)	26.3 (26)	0.075^*^; <0.001^**^; 0.046^***^
Agitated	29.3 (29)	8.1 (8)	0.0 (0)	<0.0001^*^; <0.00001^**^; 0.001^***^
Sleepiness	26.3 (26)	13.1 (13)	4.1 (4)	0.010^*^; <0.0001^**^; 0.011^***^

^*^After 3 months vs. at inclusion; ^**^After 6 months vs. at inclusion; ^***^After 6 months vs. after 3 months. NA, not applicable.

**Figure 4:**
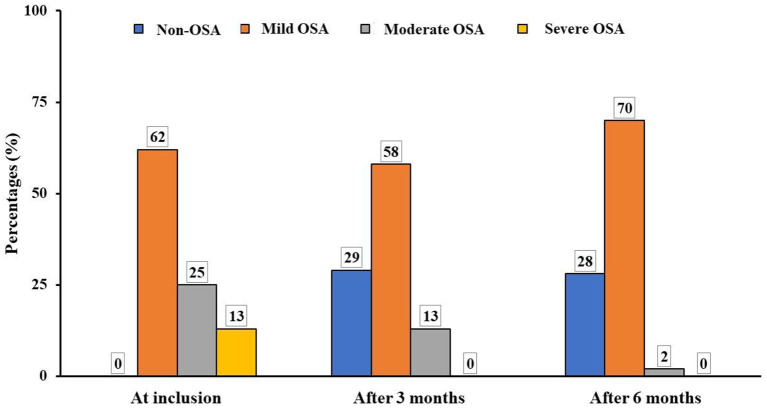
Modification of OSA severity after treatment in study subjects. OSA, obstructive sleep apnea.

The results demonstrated that the severity of OSA was significantly reduced after 3 months and 6 months of treatment by LRA. The percentage of moderate OSA was decreased from 25.3% at inclusion to 2% after 6 months. The percentage of study subjects with severe OSA was significantly reduced from 13.1 to 0.0% after being treated with LRA. The percentage of study subjects without OSA was significantly increased after being treated with LRA (0.0 vs. 28%) (Figure 4).

## Discussion

The results of the present study showed that among 139 children with asthma there was 99 patients with OSA were included in the study (Table 1). Hence, the incidence of OSA in asthmatic children was high, accounting for 71.2% (data not shown). High prevalence of OSA in asthmatic patients may be due to asthma shares the same risk factors for OSA such as allergic rhinitis. This disease increases the risk of upper airway collapse, apnea – hypopnea events, and respiratory efforts to overcome the narrowing of upper airways during sleep (4–7, 17). The average age of the study subjects was 9.3 years with an average BMI of 17.4 kg/m^2^, of which male patients accounted for 73.4%, higher than female patients 2.8 times (Table 1). Gender differences in asthma in children have also been better described and understood in recent years (18). In the 1st years of life, boys were at higher risk of asthma than girls with the proportion of asthmatic boys was almost twice of girls (18).

There are some similar clinical manifestations sharing between OSA and asthma during the night such as low quality of sleep, snoring, shortness of breath, intermittent apnea - hypopnea, frequent wake-up, difficulty falling asleep, sweating, or enuresis (19). In the present study, children with asthma who had nighttime symptoms such as snoring (61.6%), difficulty for falling asleep (45.5%), disturbed sleep (76.8%), and frequent wake-up (34.8%) increased the risk of OSA (Table 2). The present study also showed that there was a significant correlation between BMI and snoring index (*R* = 0.189 and *P* = 0.027; data not shown). Other symptoms such as difficulty of breathing during sleep, frequent wake-up, sweating in children with asthma did not increase the risk of OSA (Table 2). It suggests that a difficulty of breathing during sleep and other symptoms might be related to the characteristics of asthma at night rather than OSA (20).

Children with OSA and without asthma often present with snoring during sleep, shortness of breath, or may not be able to fall in sleep or often wake up (1). It reduces the quality of children' sleep and does not help them to have a refresh after sleep (1). For older children, sleep disturbance might increase the risk of personality and/or behavior disorders, physical or mental development delay that may affect their learning, memorizing and health issues (8). In the present study, the results of daytime symptoms of asthmatic children with OSA showed that 46.5% of them were irritable, 30.3% had abnormal behavior, 29.3% were agitated and 26.3% had daytime sleepiness (Table 2). This proportion was relatively high and suggests that it is very harmful if the patients did not have the proper treatment. Moreover, the present study revealed that most asthma patients had a atopy and 85.86% of them had allergic rhinitis (Table 1). Previous study reported that nearly 80% of asthmatic patients with allergic rhinitis was associated with an increased risk of OSA (21).

In children without asthma, the prevalence of OSA has been varied from 1 to 5% and it may be occurred in all ages within the highest incidence in 2–8 years (8–12%) (1–3). In comparison with children without asthma, the higher prevalence of OSA in asthmatic children suggests that this high prevalence of comorbidity could require a special attention because it can make asthma more difficult to control (1, 13). In patients with asthma, OSA plays a role as a contributor for aggravating asthma because the upper airway obstruction due to OSA in nocturnal asthmatic patients might be associated with sleep disturbances and daytime sleepiness (19, 20). However, the correlation between asthmatic subjects with OSA and asthma severity, treatment adherence, and asthma control remains controversial (22, 23). In children, based on the result of the recent systematic review, Sánchez et al. revealed that children with asthma were more likely to develop habitual snoring and OSA, and children with sleep disordered breathing were more likely to develop asthma (22). This result is similar with our previous study (17).

The present study could not find out the significant correlation between FEV_1_ and AHI index (Figure 2B). It might suggest that the degree of bronchial obstruction is not related to the severity of OSA in asthmatic children with OSA. Therefore, the use of spirometry alone is not useful for screening patients with OSA. This feature might be due to the pathogenesis of OSA is mainly related to the obstruction of upper airways (1, 3). The present study also demonstrated that asthmatic patients with OSA had the high levels of exhaled NO (bronchial and nasal F_E_NO and C_A_NO) for children (Table 1). Interestingly, there were only the weak significant correlations between bronchial and nasal F_E_NO with AHI (Figures 2C,D). Although the level of exhaled NO might be increased in adult with OSA (24), in children with asthma, the high level of exhaled NO has been considered as a marker of allergic inflammation due to eosinophilia that has not been well controlled by ICS and requires increased ICS dose as suggested by our previous study (14, 25). However, if clinical symptoms of asthma are well-controlled and confirmed by ACT scores, it may suggest that high level of F_E_NO in asthmatic patients with OSA might be contributed by airway inflammation due to oxidative stress (25).

The present study also demonstrated that after 6 months of treatment, the percentage of intermittent asthma patients increased significantly compared to at inclusion; notably, the percentage of moderate asthmatic patients was decreased significantly (Figure 3A). Especially, after 6 months of treatment, there was no patient with severe asthma. This result suggests that the combination of ICS and LRA in asthmatic children with OSA could improve asthma severity as recommended by GINA (26). In addition, the percentage of uncontrolled asthma was also decreased after combined treatment with ICS and LRA; inversely, the percentage of well controlled asthma was increased significantly after 3 months and 6 months of treatment (Figure 3B). Consequently, the mean ACT scores were significantly increased after treatment (Table 2).

In the present study, the improvement of clinical symptoms of asthma was also confirmed by the modification of respiratory parameters, such as low FEV_1_ and PEF at inclusion compared with those higher after 3 months and 6 months of treatment (*P* < 0.05; Table 3). These results are consistent with Anandi's study on 32 asthmatic children aged 6–12 years old, with improved clinical symptoms, increased FEV_1_ and FVC values after 3 months of treatment, and PEF was increased significantly after 6 months of treatment (27). In the present study, the mean levels of bronchial F_E_NO of asthmatic children measured after 3 months and 6 months of treatment were lower than that at inclusion (*P* < 0.05; Table 3). Thus, bronchial F_E_NO <20 ppb has been recommended as the target of controlled asthma monitoring (16).

Obviously, the results of the present study showed that after 6 months of treatment with ICS and LRA there was an significant improvement of OSA symptoms in study subjects, especially for nocturnal symptoms, such as snoring and difficulty for falling asleep which were improved after 3 months and 6 months of treatment (Table 4). Other symptoms at night were also improved remarkably, including difficulty of breathing during sleep and frequent awake (Table 4). Other daytime symptoms were also improved significantly after 3 months and 6 months of treatment. For instance, abnormal behavior and daytime sleepiness were decreased sharply after 6 months (Table 4). This result is similar with previous published studies (10, 12). In addition, in the present study, the results of RPG confirmed OSA severity was decreased significantly after 3 months and improved after 6 months of treatment with ICS and LRA for asthma (Figure 4). Definitely, the percentage of children with moderate or severe OSA was significantly reduced after the combined treatment; it was similar to previous studies (1, 28).

The present study showed that after giving the treatment with LRA, there was no case with side effects was detected and required the treatment discontinuation. Hence, this treatment could be considered as an effective therapy for improving both clinical symptoms and RPG (10, 12). The results of the present study after 6 months confirmed the treatment of asthma with ICS in combination with LRA had both effective role in the asthma control and in the improvement of the symptoms and severity of OSA. Finally, the main limitations of the present study have been related to the limited number of study population, the short duration for patients' follow-up (only 6 months), and the lack of controlled asthmatic group without OSA. Therefore, the long-term follow-up with large scale study population and randomized controlled study could be necessary for evaluating the first choice of ICS combined with LRA in the treatment of asthmatic children with suggested OSA symptoms for a personalized therapy in the future of asthma management.

## Conclusion

OSA is a common comorbidity in children with asthma. Asthmatic children with OSA usually have the symptoms at night and its consequences during the day. The presence of snoring, high exhaled NO level, and dyspnea during sleep in asthmatic children may be associated with a higher risk of OSA. The treatment with leukotriene receptor antagonists, in combination with inhaled corticosteroids according to GINA recommendations, for children with asthma can improve both asthma control and symptoms of OSA in asthmatic patients with comorbid OSA.

## Data availability statement

The raw data supporting the conclusions of this article will be made available by the authors, without undue reservation.

## Ethics statement

The studies involving human participants were reviewed and approved by Ethics Council in Biomedical Research of Hanoi Medical University within the Decision No. 187/HDDD/DHY-HN. Written informed consent to participate in this study was provided by the participants' legal guardian/next of kin.

## Author contributions

SD-Q, YN-H, LN-N-Q, MN-T-P, HN-T-B, HL-T-M, and TN-T-D: conceptualization, validation, and writing—original draft preparation. SD-Q, YN-H, LN-N-Q, and TN-T-D: methodology and writing—review and editing. SD-Q, YN-H, and LN-N-Q: software. SD-Q, YN-H, LN-N-Q, HL-T-M, and TN-T-D: formal analysis. All authors contributed to the article and approved the submitted version.
